# Hyperthermal Reactions in DNA Triggered by 1–20 eV Electrons: Absolute Cross Sections for Crosslinks, Strand Breaks, Clustered Damages and Base Modifications

**DOI:** 10.3390/ijms26094057

**Published:** 2025-04-25

**Authors:** Yanfang Dong, Xin Huang, Wenlu Zhang, Yu Shao, Pierre Cloutier, Yi Zheng, Léon Sanche

**Affiliations:** 1 College of Basic Medicine and Forensic Medicine, Henan University of Science and Technology, Luoyang 471000, China; yangf_dong@163.com (Y.D.); z907733270@163.com (W.Z.); 2 State Key Laboratory of Photocatalysis on Energy and Environment, Faculty of Chemistry, Fuzhou University, Fuzhou 350116, China; hx614352503@163.com (X.H.); shaoyu@fzu.edu.cn (Y.S.); yi.zheng@usherbrooke.ca (Y.Z.); 3 Department of Medical Imaging and Radiation Sciences, Faculty of Medicine and Health Sciences, Université de Sherbrooke, Sherbrooke, QC J1H 5N4, Canada; pierre.cloutier@usherbrooke.ca

**Keywords:** cross sections, low-energy electrons, DNA lesions, radiation damage, Monte Carlo

## Abstract

Absolute cross sections (ACSs) are needed to estimate cellular damage induced by high-energy radiation (HER). Low-energy electrons (LEEs), which are the most numerous secondary particles generated by HER, can trigger hyperthermal reactions in DNA. ACSs for such reactions are essential input parameters to calculate radiobiological effectiveness, particularly in targeted radiotherapy. Using a mathematical model, we generate ACSs from effective damage yields induced by LEE impact on 3197 base-pair plasmid DNA films. Direct or enzyme-revealed conformational damages, quantified by electrophoresis, provide the first complete set of ACSs for inducing crosslinks, double-strand breaks (DSBs), single-strand breaks, base-damage-related crosslinks, non-DSB clustered damages (NDCDs), and isolated base damages. These ACSs are generated across the 1–20 eV range, at one eV intervals. They exhibit a strong energy dependence with maximum values at 10 eV of 3.7 ± 0.8, 3.5 ± 0.6, 45.4 ± 4.1, 2.9 ± 1.1, 5.1 ± 1.4, and 54.0 ± 16.4 × 10^−15^ cm^2^, respectively. ACSs for DSBs, NDCDs, and crosslinks clearly indicate that lesions threatening cell function and genetic stability can be generated by a single LEE. At 5 and 10 eV, total damage ACSs are 63% and 80% larger, respectively, than those previously determined for the same plasmids bound to arginine, a constituent of histones protecting DNA.

## 1. Introduction

In molecular solids and liquids, cross sections (CSs) are essential parameters to quantify the magnitude of reactions triggered by low-energy (0–30 eV) electrons (LEEs) [1]. Such CSs can find applications in numerous condensed-phase electron- or photoelectron-induced processes at surfaces [2,3,4], interfaces [5,6] or within solids [7,8,9]. More specifically, LEEs are involved in dielectric aging and breakdown of insulators [10], the fabrication of nanostructures [11,12], plasmon and plasma chemistry [13,14], LEE microscopy [15], and tunable chemistry at surfaces [16,17,18,19,20,21]. LEEs also play a role as secondary electrons (SEs), which are the most abundant species produced by high-energy radiation (HER) [22,23]. CSs for processes induced by LEEs can thus also find specific applications in HER-related fields. These include planetary science [24], astrochemistry [25,26,27,28], nanolithography [8,29], electron microscopies [30,31], radiation chemistry [32,33,34,35], radiobiology [36,37], space travel [38], nuclear waste management [39,40], radiation protection [41], surface processing [42], and radiotherapy [43]. In other words, LEE CSs represent an important parameter in the radiation sciences [44,45], particularly to quantify the early sequence of events immediately following the initial ionization induced by HER in condensed media [1].

In living organisms, when HER interacts with a cell, about 3 × 10^4^ LEEs per MeV of deposited energy are initially generated [22,36]. Along their paths, LEEs can produce dissociative electronically excited molecules that can produce reactive radicals. A LEE can also temporarily attach to a molecule, forming a transient anion (TA), which can decay via dissociative electron attachment (DEA) or autoionization. Via autoionization, the molecule can also be left in a dissociative electronically excited state. Thus, both processes can produce highly reactive radicals [46]. As they lose their energy in cells, LEEs and the species they produce interact with small biomolecules (e.g., H_2_O) and with much more complex molecules, such as DNA [46]. The initial hyperthermal reactions and the following slower thermal reactions modify the molecular content of irradiated cells. Complex DNA damage, which is hard to repair, can induce the loss of cell functionality, apoptosis, or mutations. Knowledge of CSs for reactions triggered by LEEs and the ensuing DNA lesions are therefore crucial to understand and model the biological effects of radiation. Since at low energies, electron interactions are highly sensitive to the environment [47], biologically relevant LEE CSs (i.e., probabilities of a given LEE event, reaction, or biomolecular damage) should not be generated from gas-phase data [1,48]. Since radiation interacts with matter stochastically, Monte Carlo (MC) codes, with their probabilistic models, appear as the preferred tool to use such LEE CSs to provide a detailed description of all events and species produced by the absorption of primary high-energy radiation in biological tissue [49,50,51,52,53,54,55,56]. The inclusion of LEE CSs in MC codes is discussed in Section 2.

Numerous experiments have measured, under the same conditions, effective yields for 5-monolayer plasmid films exposed in vacuum to a monoenergetic LEE beam of fixed energies [57,58,59,60,61]. After the bombardment, the samples were analyzed by electrophoresis. Only some types of damages were identified and quantified as effective yields and then transformed into absolute cross sections (ACSs) [59,60,61]. Moreover, since ACSs were provided only at a few specific energies [59,60,61], they may not provide a sufficient database for MC codes.

In the present article, we provide the first complete set of absolute LEE CSs for all measurable damages induced in plasmid DNA by 1–20 eV electrons. Recorded at one eV intervals, the database includes ACSs to induce single-strand breaks (SSBs), isolated base damages (BDs), inter-duplex crosslinks (CLs), base-damage-related CLs (BD-CLs), double-strand breaks (DSBs), and other clustered lesions referred to as non-DSB cluster damages (NDCDs). The plasmids are extracted from bacteria. They correspond to the type of double-helix DNA generally found in human mitochondria and can serve as a suitable model of genomic DNA [62]. The present plasmids consist of 3197 base pairs, which is certainly sufficient to include the quantum behavior of LEEs in the measured CSs. The ACSs are generated through an extension of the survival model developed by Rezaee et al. [63] and the effective quantum yields measured by Dong et al. [57,64]. The extended mathematical model is described in Section 3. The absolute values of the 1–20 eV energy dependence of the CSs generated from this model are discussed in Section 4.1, and they are compared to those previously measured at 5, 6, and 10 eV in the same plasmids bound to the amino acid arginine in Section 4.2 [65]. In Section 4.3, we describe the hyperthermal reactions triggered by LEEs, whose ACSs are provided in this paper. Abbreviations defined in the text are listed at the end.

## 2. Incorporating LEE Cross Sections into Monte Carlo Codes

MC codes can describe the event-by-event production of all species produced and the subsequent reactions in irradiated biological media [53,55,56,66]. Since water often acts as a surrogate for the cell, MC simulations in water have received considerable attention [67,68,69,70,71,72,73,74]. However, the lack of accurate LEE CSs for liquid water has been a considerable source of uncertainty in the outcome of simulations, and the use of existing amorphous ice CSs as a replacement has been discussed for decades. Many authors have therefore relied on a theoretical description of the LEE scattering, often combined with experimental results [54,75,76,77,78,79,80,81]. Recently, Signorell compared experimental data from photoelectron scattering in *liquid water* with corresponding MC calculations of the transport equation using amorphous ice CSs [82]. The results strongly suggest that amorphous ice CSs, with the extension up to a few hundred eVs, provide the most reliable values for liquid water [83].

General-purpose MC codes are also available for simulations of electron tracks causing different types of DNA damage [46,53,70,75,84,85,86,87,88,89,90,91,92,93,94,95]. Not only ionization CSs of DNA components but also DEA CSs were incorporated into calculations to simulate the contributions of LEEs to various types of damage [75,94,95,96]. Inelastic CSs on the simulation of direct DNA strand breaks induced by LEEs were also loaded into the Geant4-DNA code to calculate DNA damage yields by applying the dielectric function optical-data treatments [51,75,92,97]. Inherent limitations persist within the various models, and the results of simulations depend to a large degree on user-defined parameters, definitions, and algorithms, including DNA modeling, dose distribution, and the DNA damage clustering algorithm [51,75,91,93,98].

Charged particle track-structure analysis by MC requires accurate total and differential CSs for all the relevant interaction mechanisms. Several sets of CSs data are required for analysis of a particular medium. Apart from amorphous ice [83], complete energy loss and elastic scattering LEE CSs *in the condensed phase* are not available for materials of interest to radiation biology. Furthermore, the simulation of electron tracks and the ensuing damage is affected by the inherent nature of the codes, which treat LEEs as classical particles interacting with matter at a specific point in space-time, i.e., the wave-like behavior of LEEs is neglected as well as the time delay caused by the formation of TAs. For water, this limitation may be more acceptable because of the short range of the electron’s coherence length [1]. However, in long DNA strands with repeated quasi-equidistant fundamental units, the wave nature of LEEs becomes a dominant factor, e.g., the 1–15 eV energy dependence of LEE elastic scattering from a 10 base-pair DNA strand undergoes destructive and constructive interference with energy, which modifies the production of reactive species via DEA [99]. We further expect “inter-spur” chemical reactions of multi-body chemical species to be affected by the quantum behavior of LEEs [100]. Nonetheless, in MC codes the fundamental DNA units (bases, sugar, and phosphate group) are usually represented as individual non-mutually interacting scattering centers to which are assigned elastic and various inelastic CSs [80,100,101,102]. As shown experimentally by Lemelin et al., LEE scattering from base and sugar moieties is highly sensitive to chemical bonding between these fundamental units [103]. Hence, both scattering of electron waves and chemical bonding between DNA constituents must be considered to obtain more realistic LEE scattering CSs and create fully integrated simulations evaluating HER-induced cellular damage [93].

A priori, CSs incorporating the quantum mechanical nature of LEEs can be generated theoretically or experimentally. They can also be combined to produce input parameters for MC codes, e.g., elastic scattering CSs are difficult to measure experimentally in the condensed phase, whereas their values can be generated by adapting gas-phase electron scattering R-matrix calculations to the DNA band structure [104]. To experimentally determine the contribution of LEEs to DNA damage, the CSs must be determined from high-resolution electron scattering from a biological form of DNA of sufficient length to incorporate the inherent multitude of quantum mechanical phenomena. Moreover, the set of CSs provided must be sufficiently complete to cover any significant damage disturbing cellular functionality resulting from single or multiple events. By incorporating such CSs, MC simulations are expected to play an increasing role in the radiobiological applications of HER, including the improvement of radiotherapy modalities, particularly concomitant chemoradiation therapy [105] and targeted radionuclide therapy [106,107]. For example, in the latter modality, the radionuclide decays mainly via the ejection of Auger electrons, with energies of a few hundred eV and ranges of 10–30 nm, i.e., a high density of LEEs is produced in the vicinity of the target. Hence, calculation of biological effectiveness and the detail of deposited energy becomes strongly dependent on absolute LEE scattering CSs. More generally, when considering damage and dose heterogeneities within the cell nucleus, chromosomes, or DNA, CSs for relevant biomolecules must be evaluated to link more directly the distributions of the lesions to the radiobiological effectiveness in any type of HER treatment or imaging modality.

## 3. Mathematical Model to Generate Absolute DNA Damage Cross Sections from Effective Yields

In LEE beam experiments, plasmids deposited as a thin layer (10–20 nm) on a metal substrate can be bombarded at a precise energy in ultrahigh vacuum. Afterwards, the damage yields are measured outside the vacuum by electrophoresis [108]. The effective yields (*Y_eff_*) are extrapolated to zero LEE fluence by recording the percentage of the total yields for each specific damage as a function of fluence. The latter is expressed as the number of incident electrons divided by the bombarded area. The linearity of the fluence-response curves near zero fluence ensures that the measured damage is induced by a single electron and hence can serve to determine ACSs. Examples of fluence-response curves, recorded with 10 eV electrons, for all damages considered in this paper are provided in Figure S1 of the Supplementary Materials. Measured effective yields can be converted to ACSs by applying an extension of the mathematical model previously described by Rezaee et al. [63]. The extension allows generating ACSs for lesions other than those producing conformational changes in the plasmids. The salient features of the extended framework are summarized in this section.

The total percentage of initial targets $P\left(t\right)$ left intact on the substrate after exposure during a time *t* to the LEE beam of energy E can be expressed as

(1) $$P\left(t\right)=P_{0}e^{-J\sigma_{eff}\left(E\right)t}\;\;\;$$

where $P_{0}$ is the percentage of intact molecules at *t* = 0, and *J* is a uniform incident LEE current density. In this equation, $\sigma_{eff}$(E) becomes the total effective CS to damage initially intact molecules in the film with electrons of energy E. This CS is termed effective because it depends on film thickness and charging from the LEE beam, as well as the influence of the metal substrate [108]. $\sigma_{eff}$(E) is contingent upon these conditions and hence cannot be transported into other situations or used as input CSs in MC simulations. For sufficiently thick DNA films of the order of the electron’s thermalization distance (~10 nm) [109], the effects of the metal substrate can be neglected, but then charges accumulate as a function of time in the film. Furthermore, the amount of damaged DNA in the film depends on thickness, since the damage diminishes with the penetration distance of the electron beam.

To eliminate the effects of charging, fluence-response curves are recorded at low current surface densities and extrapolated to zero fluence [63]. At small electron fluences, the total effective CS ($\sigma_{eff}$) reduces to

(2) $${\;\sigma }_{eff}=\frac{P^{\prime }(0)}{P_{0}J}=Y_{eff}\;$$

where $P’\left(0\right)\;is\;the\;slope\;of\;P\left(t\right)\;at\;t=0\;and\;Y_{eff}\;$ the total effective yield represented as the percentage of total DNA damage per unit of fluence measured near zero fluence. According to the model of Rezaee et al. [63], after LEE bombardment during time *t* of a DNA film of thickness *h*, *P*(t) can also be expressed as

(3) $$P\left(t\right)=P_{0}\frac{1}{h}\int_{0}^{h}e^{-\sigma J\tau (1-e^{-\frac{t}{\tau }})e^{-\frac{x}{\lambda }}}dx$$

where λ is the attenuation length, *σ* the ACS, and *τ* the film charging time constant [110,111]. From simulations of *P*(*t*) vs. fluence with different values of the parameters, the slopes of the *P*(*t*) vs. electron fluence curves for different *τ* at *t =* 0 were found to be the same, i.e., the initial (*t = 0*) rate of decrease in the concentration of the initial target molecules is independent of film charging [63]. Therefore, for sufficiently short irradiation times, charging should have a minimal effect on the linear slope of fluence-response curves. Under this condition, the initial slope *P*’(0) of *P*(*t*) vs. electron fluence curves is given by

(4) $$P^{\prime }\left(0\right)=-P_{0}\sigma J\left(\frac{\lambda }{h}\right)\left(1-e^{-\frac{h}{\lambda }}\right)$$

The average value of *λ* is determined experimentally from the ratios of initial slopes of fluence-response curves for the loss of the supercoiled configuration, usually for three film thicknesses [60,61,63]. From the expressions in the brackets of (4), a penetration factor *f* is defined as

(5) $$f=\frac{\lambda }{h}\left(1-e^{-\frac{h}{\lambda }}\right)$$

and from Equations (2), (4) and (5), the ACS can be expressed as

(6) $$\;\;\sigma =\frac{P’\left(0\right)}{P_{0}Jf}=\frac{\sigma_{eff}}{f}$$

$\sigma_{eff}$ includes the yields of conformational damages directly measured by electrophoresis as SSBs, DSBs, and CLs, as well as any other damage that reduces the integrity of the initial DNA molecules but is not detected (ND) by electrophoresis. Without enzyme treatment, analysis by electrophoresis can only detect conformational damage changes consisting of the circular (SSBs) and linear (DSBs) configurations and two plasmids bound together (crosslinks). The ACSs, $\sigma_{SSB},\sigma_{DSB},\sigma_{CL}\;and\;\sigma_{ND}\;$ can be, respectively, assigned to these damages. Considering the linearity of Eq 6, we can write the total ACS as

(7) $$\sigma_{total}=\sigma_{SSB}+\sigma_{DSB}+\sigma_{CL}{+\sigma }_{ND}=\frac{P^{\prime }{\left(0\right)}_{SSB}+P^{\prime }{\left(0\right)}_{DSB}+P^{\prime }{\left(0\right)}_{CL}+P^{\prime }{\left(0\right)}_{ND}}{P_{0}Jf}$$

which allows equating each $\;\sigma_{SSB},\sigma_{DSB},\sigma_{CL}\;and\;\sigma_{ND}$ to the corresponding slopes at zero fluence and thus generating individual ACSs.

To measure base-damage-related lesions, we use enzymes that can specifically recognize and remove damaged pyrimidine and purine bases within the plasmid and convert BDs into strand breaks by hydrolysis of the glycosidic bonds [112,113]. The additional strand breaks and crosslinks created by the enzyme treatment can be detected by gel electrophoresis. BDs are converted to SSBs or inter-duplex CLs if the open bond reacts with a nearby plasmid, i.e., a BD-CLs. If the enzyme-treated plasmid contains a SSB or a BD in the opposite strand within 20 base pairs, NDCDs can be identified as an increase in the yields of DSBs. Thus, the additional yields due to enzyme treatment appear in the gel as SSBs, DSBs, and CLs. The ACS calculated from these additional yields can be added to Equation (7). Another term is also added ($\sigma_{OND})\;$ to account for the ACS of the sum of any other non-detected (OND) damages. Thus, $\sigma_{ND}$ can be written as

(8) $$\;\sigma_{ND}=\sigma_{BD}+\sigma_{BD-CLs}+\sigma_{NDCD}+\sigma_{OND}=\frac{P^{\prime }{\left(0\right)}_{BD}+P^{\prime }{\left(0\right)}_{BD-CLs}+P^{\prime }{\left(0\right)}_{NDCD}+P^{\prime }{\left(0\right)}_{OND}\;}{P_{0}Jf}$$

We note that this model does not require knowledge of the total effective yield or ACSs to determine individual ACSs values from the experimentally measured effective yields of different lesions. Furthermore, since σ in Equation (6) is an ACS, the ratio $\frac{Y_{eff}}{f}$ is transportable from one experiment to another, as long as the corresponding measurements are performed under the same conditions with similar films.

## 4. Results and Discussion

### 4.1. ACSs for DNA Damages Induced by 1–20 eV LEEs

So far, LEE ACSs for DNA damage have been measured at fixed energies, typically at 10 eV. ACSs recorded at 10 eV for the loss of the supercoiled (LS) configuration are listed in Table S1 of the Supplementary Materials. Results within the widest energy range were reported by Chen et al. [60]. They applied the molecular survival model [63] to generate ACSs for the loss of supercoiled (LS) configuration and formation of SSBs, CLs, and DSBs induced by electrons of energies 4.6, 5.6, 9.6, and 14.6 eV. The ACSs of LS induced by 10-eV electrons was 51 ± 1 ×10^−15^ cm^2^ [60], whereas the effective CS was 13.0 ± 1.2 × 10^−15^ cm^2^. The results demonstrated that the penetration factor *f* depends on the film thickness, but it is independent of electron energy [63]. Thus, by applying Equation (6), we can deduce *f* to be 0.25 ± 0.02, which can be applied to estimate the ACSs for a larger variety of DNA damages induced by electrons in the range 1–20 eV from effective yields ${(Y}_{eff})$ previously measured by Dong et al. [57,64] in this energy range. The resulting ACSs for all measured $Y_{eff}$ are listed in Table 1.

**Figure 1:**
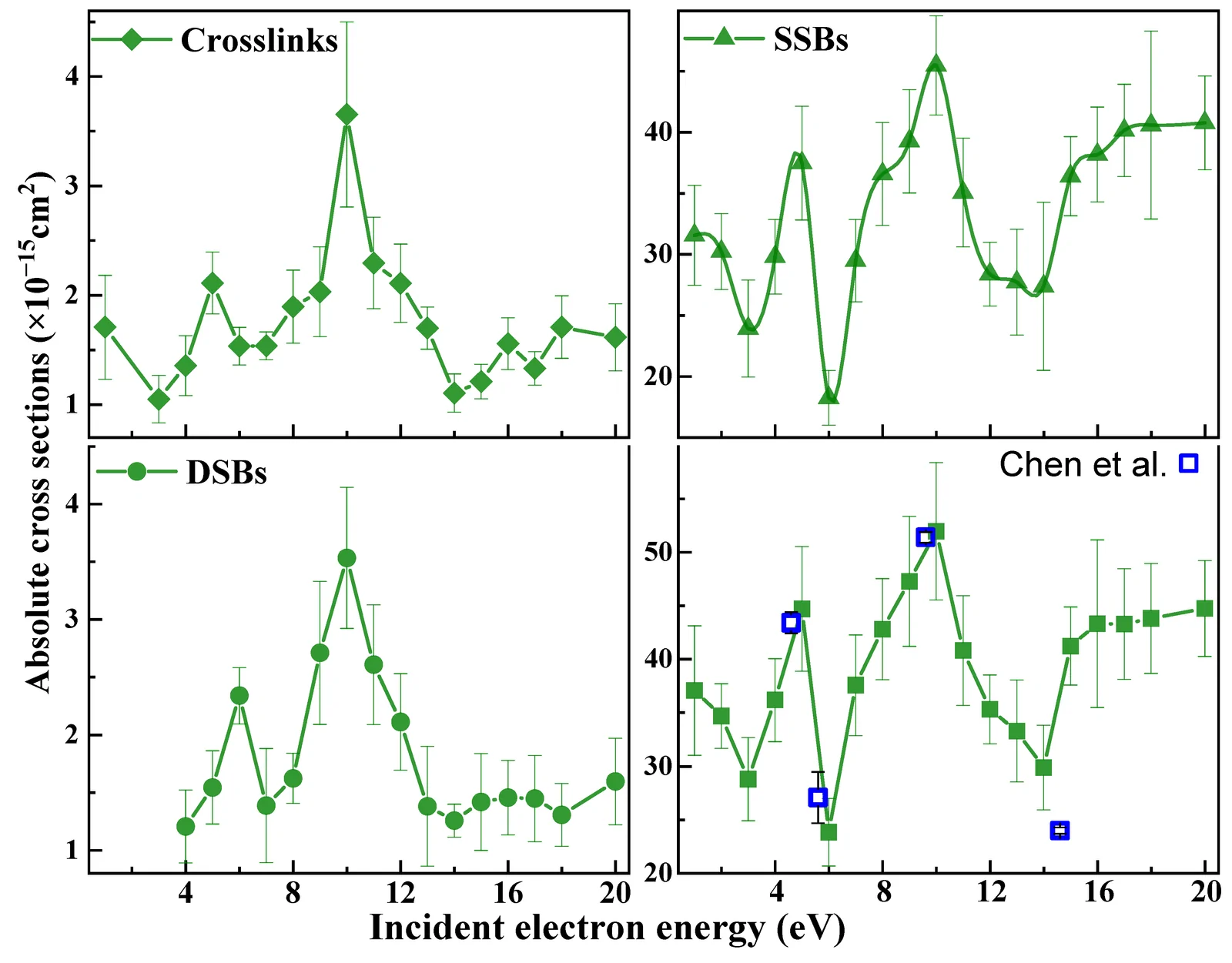
ACSs for CL (◆), SSB (▲), DSB (●), and LS (■) induced in 3197 base-pair plasmid DNA by 1−20 eV electrons. The values were generated from effective yields with *f* = 0.25 ± 0.02 [104,111]. The error bars arise from the experimental standard deviations. The open points (**□**) are the average ACSs for the loss of the supercoiled configuration of the same plasmids under identical conditions taken from the work of Chen et al. [60]. The standard deviations in the results of Chen et al. are of the order of 11 %. Data points from both experiments agree well within experimental errors.

Figure 1 illustrates graphically the results of Table 1 for prompt CLs, SSBs, DSBs, and LS as a function of electron energy. The ACSs of LS agree very well with the open blue squares, which were calculated from $Y_{eff}$ values measured by Chen et al. [60], thus corroborating the present ACSs and assessing the effectiveness of the approach. The ACS of LS and SSBs are at least one order of magnitude larger than those of DSBs and CLs. As expected, the ACSs for LS are similar to those of SSBs without enzyme treatment, since in this case, DNA in the initial supercoiled configuration is mainly converted to the circular form caused by an SSB [57].

**Figure 2:**
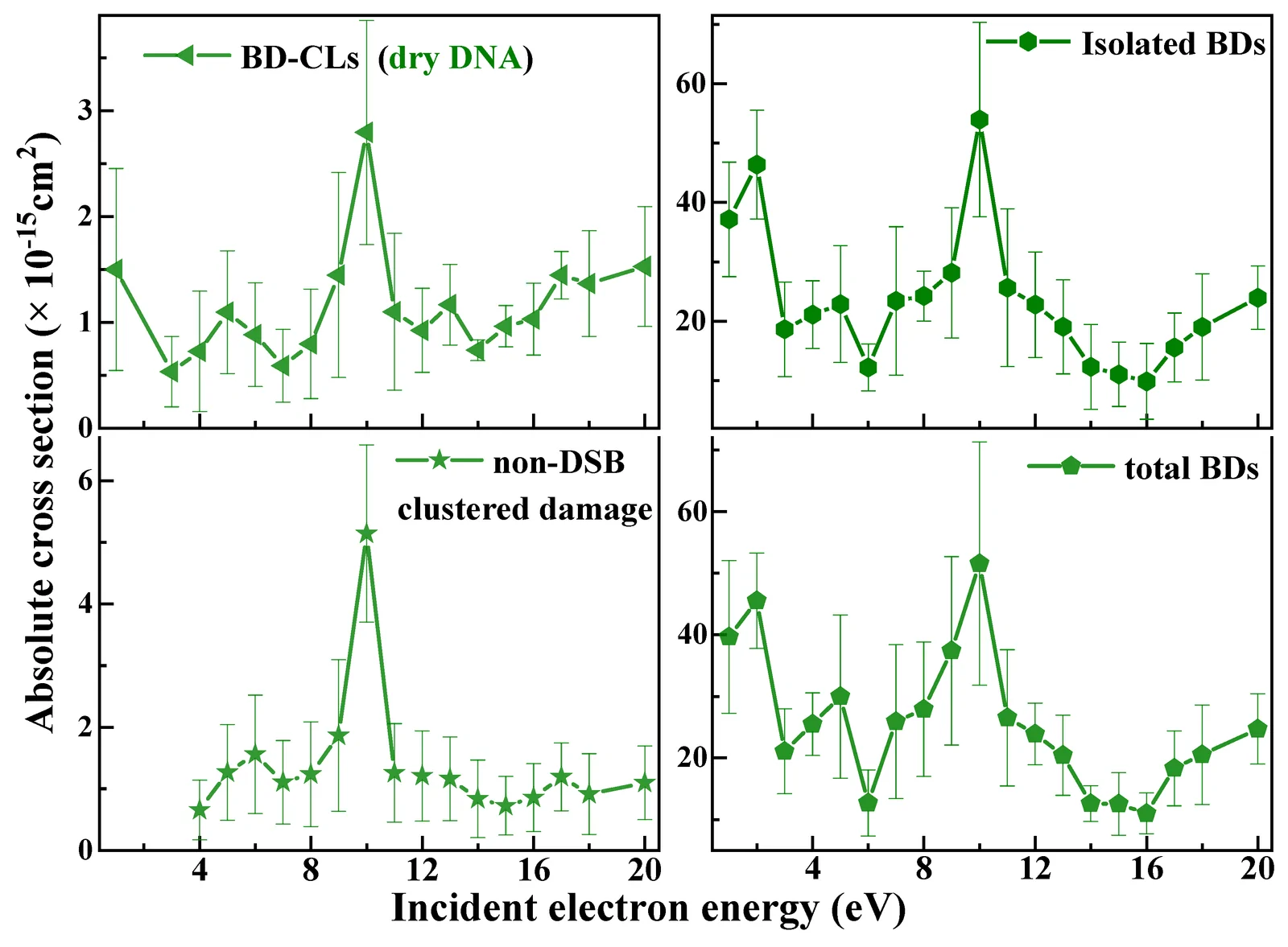
The ACSs of BD−CLs (◀), isolated BD (▶), non-DSB cluster damage (★), and total BDs (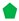) induced by 1−20 eV electrons in 3197 base-pair plasmid DNA. The absolute values are generated from effective yields and a penetration factor *f* of 0.25 ± 0.02, which is obtained from the ratio of the effective cross section to the ACS (Equation (6)) for loss of the supercoiled configuration induced by 10 eV electron impact on 10, 15, and 20 nm plasmid DNA films [57,64]. The error bars arise principally from the standard deviations in the measured effective yields.

From BD revealed by base excision repair endonuclease (Nth and Fpg) treatments, we report in Table 1 the first determination of BD-related ACSs for duplex DNA. Thus, previous measurements of only conformational damages underestimated the total damage CSs [60,61,63]. The effective CSs were converted to ACSs using *f* = 0.25 ± 0.02 and the linearity of Equation (8). The electron energy dependence of the ACSs for BD-related lesions, including BD-CLs, isolated BDs, non-DSB clustered damages, and total BDs, are shown in Figure 2. The ACSs for isolated BDs being close to those of total BDs indicate that the former constitute the most numerous base-related lesions. The detected NDCDs correspond to two BDs on opposite strands or a BD with a strand break on the adjacent strand, both within 20 base pairs. The ACS of NDCDs is at least one order of magnitude smaller than that of isolated BDs. Clustered DNA lesions (e.g., DSBs and NDCDs) cannot be effectively repaired by a single mechanism, such as base excision repair [113], homologous recombination [114] or non-homologous end joining [115], which in turn can lead to genetic aberrations, mutations, and chromosomal instability, thus affecting cell function and promoting aging, cancer, and inflammation [116]. The relatively high ACSs to induce cluster damage with a single electron demonstrate the significant role of LEEs in radiation-induced DNA damage.

From Figure 1 and Figure 2, the ACSs for SSBs, CLs, BD−CLs, isolated BDs, and total BDs exhibit maxima appearing at 5 and 10 eV and a rise in the 1–2 eV region. ACSs of DSBs and NDCDs have maxima at 6 and 10 eV. These features were previously observed in the electron energy dependence of the effective yields (i.e., the yield functions) [57,64]. Since below 4 eV, cluster damage is not observed in LEE-DNA experiments [57,64], the 1–2 eV peak in the ACSs of SSB, BD, and CL can be assigned to the decay of shape resonances via DEA, previously observed at 0.8 and 2.2 eV in the SSB yield function [117]. The two maxima in all ACSs arise from the formation of TAs, most likely through the initial capture of an electron by the electron affinity of an excited electronic state of a base, which subsequently decays into dissociative channels immediately or after electron transfer to another subunit [118]. The opening of a bond in DNA can form an inter-duplex crosslink, referred to as CL or BD-CL if it arises from base damage. More details are provided in Section 4.3 on the mechanisms creating all types of lesions. The increase by one eV from 5 eV for the single-lesions to 6 eV for the cluster-lesion ACSs may be related to the higher energy required to break at least two bonds in producing a cluster lesion. The rise in the ACSs of single damages from 14 or 16 eV to 20 eV is probably due to ionization, which would produce resonant lower-energy electrons. The overall signal below 16 eV in all figures arises from the overlap of the TA peaks and possibly DEA from less intense TAs that cannot be resolved in these experiments.

### 4.2. Comparison of Damage ACSs for DNA to Those of Arg-DNA Complexes

Within the genome, DNA is wrapped around histone proteins, which protect the molecule against radiation damage [119]. Recently, Wang et al. investigated 5- and 10-eV electron interactions with arginine (Arg), a major component of histone proteins [65]. The same plasmids as those investigated in the present experiments were intercalated with arginine to form films of 7, 12, and 17 nm to be irradiated in a vacuum. The effective damage yields were measured by electrophoresis, and the penetration length λ of Equation (4) was determined from the variation in the effective yields with film thickness. Applying the survival model of Rezaee et al. [63], the ACSs for all types of measurable DNA damage by electrophoresis were generated from the effective yields. The ACSs reported for Arg-DNA complexes induced by 5 and 10 eV electrons are listed at the bottom of Table 1 [65]. Except for CLs and BD-CLs at 10 eV, the ACSs are smaller for Arg-DNA complexes than the present ones. Since CLs are low-yield products, these comparisons indicate that arginine protects DNA from LEE-induced damage. In fact, the present ACSs for the total damage are approximately 63% and 80% larger at 5 and 10 eV, respectively, than those previously determined for the same plasmids bound to arginine. The protection factors (PFs), calculated from the ratios of ACSs for DNA to those of Arg-DNA (σ_DNA_/σ_Arg−DNA_) from the present data, are compared to those reported by Wang et al. in Table S2. The present PFs for DSBs, SSBs, LS, NDCDs, isolated BDs, total BDs, and total DNA damage agree well with those previously reported.

In the energy range 1–15 eV, which is dominated by TAs, the magnitude of the PFs relies essentially on the modification of electron attachment probability to the bases, the electron transfer rate to the phosphate group, and the lifetime of TAs formed on that group and on the bases [46,65]. Since these parameters are interconnected, it is difficult to quantify how individual elements change the magnitude of the ACSs when arginine binds to DNA, as shown in Figure S2 [120,121]. We note, however, that the most prominent binding resides between NH_3_^+^ of arginine and PO_4_^2−^ in DNA. Displacement of a negative charge from the phosphate toward the NH_3_^+^ group could reduce electron attachment probability to the phosphate subunit and the electron transfer rate from the base to the 3′C and 5′C positions of the sugar−phosphate bonds due to modification of potential energy curve crossing between the base and phosphate orbital configurations [58,122]. Such modification would protect DNA, and since this is the most occupied binding site, the PF for SSBs would be the highest, as clearly observed experimentally (Table S2); e.g., PFs for SSBs are 4.5 ± 0.7 and 3.7 ± 0.5, whereas for the sum of all damages, they are 1.6 ± 0.3 and 1.8 ± 0.4 at 5 and 10 eV, respectively.

### 4.3. Hyperthermal Reactions in DNA Triggered by 1–20 eV Electrons

Whereas most chemical reactions occur at room temperature, those induced by HER may happen at much higher temperatures, including the hyperthermal reactions induced by LEEs in DNA, e.g., considering that at room temperature (298 K) the motion of matter corresponds to about 0.026 eV average energy, a reaction outside thermal equilibrium induced by a TA formed at 1 eV can be considered to be initiated at a temperature of 11,462 K. We explain below how such reactions damage DNA.

LEE scattering from molecules can be considered as resonant or direct. In direct scattering, the time-dependent amplitude of the wave function of the projectile LEE does not increase significantly close to a target molecule. In this case, insight into the physical phenomenon can be obtained via analysis of the interaction potential, which generally shows that at low energies the magnitude of inelastic scattering and damage CSs is small [123]. On the other hand, if the electron spends a relatively long time with the target compared to the direct scattering time, a TA is formed. Temporary electron localization results in the extra electron occupying a previously unfilled orbital of the molecule with lifetimes ranging from a few femtoseconds to a picosecond [124]. In a complex and long molecule like DNA, the incoming electron interacts with a limited number of fundamental units and usually localizes on one of them to form a TA [99]. The TA necessarily perturbs surrounding orbitals, thus leading to an impressive number of decay channels that can produce specific damages, such as those previously enumerated. Whereas direct scattering can produce hyperthermal reactions via the production of reactive and dissociative electronically excited states within DNA, TAs, by decaying into a multitude of inelastic channels, can trigger hyperthermal reactions not possible via direct scattering. Moreover, since direct scattering is expected to be small at low energy, the main mechanisms responsible for the magnitude of the ACSs below 15 eV listed in Table 1 can be mainly attributed to the formation of TAs. Substantially, these resonances give rise to the peaks around 5–6 eV and 10 eV, as well as to the rise around 1–2 eV shown in Figure 1 and Figure 2, as explained in the previous section.

**Figure 3:**
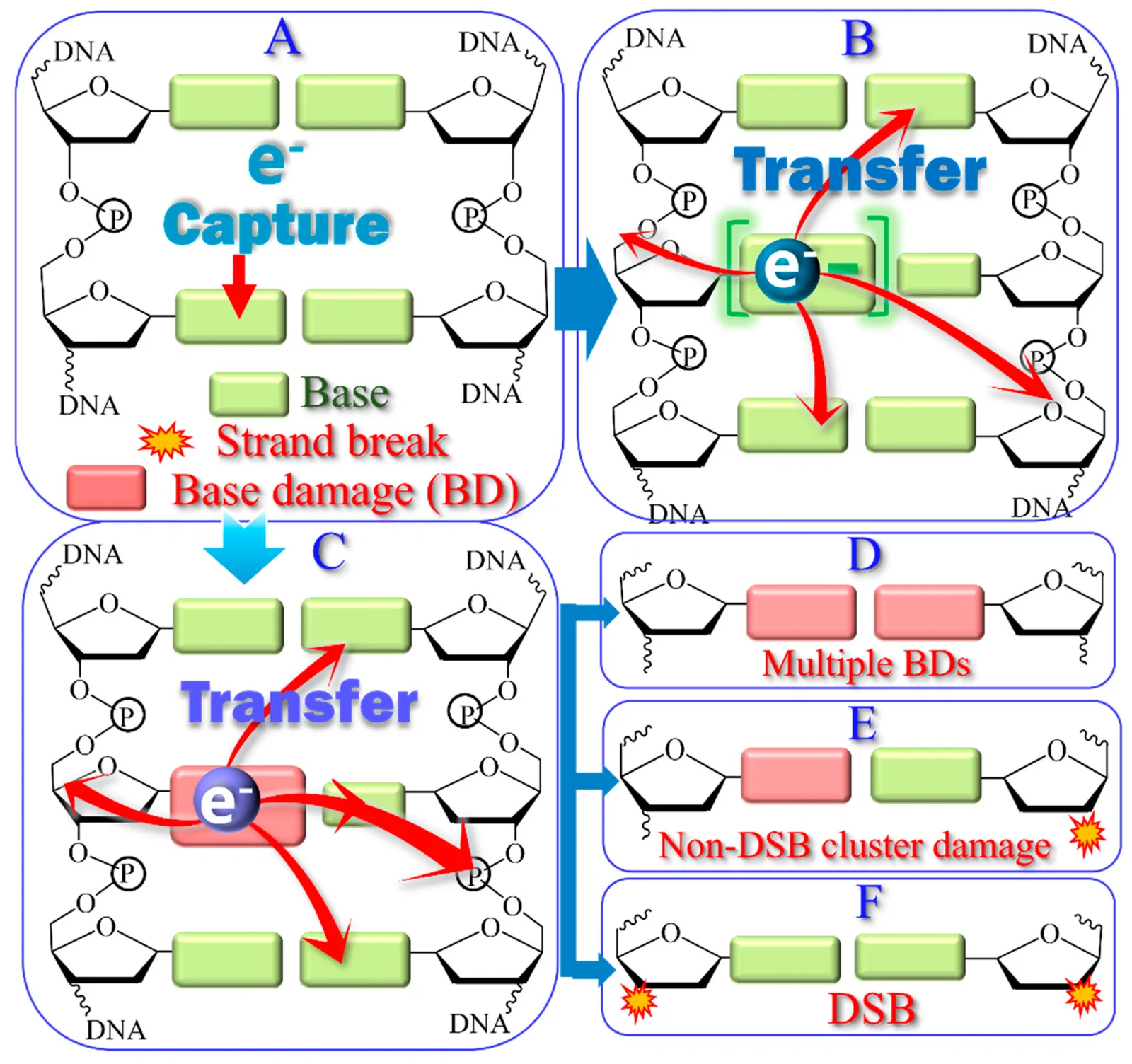
Scheme of hyperthermal reactions in DNA triggered by LEEs via shape and core-excited resonances. (**A**) The electron is captured by a base. (**B**) It stays on the base or transfers to another base or a phosphate group. If at any position in B the TA dissociates, a single base damage or strand break is created. In (**C**), the captured electron produces a dissociative electronically excited state, which damages the base. If afterwards the electron transfers to another base or a phosphate group, cluster damages (**D**–**F**) become possible via DEA.

The major hyperthermal reactions triggered by LEEs below 15 eV in a double-stranded DNA chain are shown schematically in Figure 3. Frame A on the upper left illustrates the formation of a TA by temporary electron binding to a base, which constitutes the most likely electron capture process [58,122,125]. This capture can occur primarily in two ways: (1) the electron temporarily occupies a previously unfilled orbital of the base, forming a shape resonance [122], or (2) the electron uses a portion of the added kinetic energy from the charge-induced polarization to excite electronically the base and gets trapped by the electron affinity of the excited state. This type of TA is referred to as a core-excited resonance. In both cases, the extra electron can transfer to an adjacent base, the phosphate group, or successively both, as shown in B. Transfer to the phosphate group via potential-energy curve-crossing usually forms a SSB through DEA [122,125]. The transfer probability necessarily influences the lifetime of the base TA and, hence, modulates the yields of BDs and SSBs to these fundamental units [126]. Whereas shape resonances cannot produce cluster lesions [117], core-excited resonances can trigger hyperthermal reactions, causing local multiple damages from an initial single-electron capture by a base. The ensuing processes are represented in frames C to F, where undamaged and damaged bases are represented by green and red rectangles, respectively. Cluster damage is possible when the transient core-excited anion autoionizes while leaving the base in a dissociative excited state, which should damage the base (C). Then, if the departing electron transfers to another base or a phosphate group, an additional lesion can occur via the formation of a shape resonance on either of these units, followed by decay into the DEA channel. The resulting double BD or NDCD on opposite strands are illustrated in frames D and E, respectively. DSBs (F) can be produced by the BDs in D or E if the base damage transforms into a SSB [127]. As recently shown theoretically, the two electrons in electronically excited orbitals of a core-excited resonance can simultaneously undergo DEA on adjacent phosphate groups while leaving the “core” (i.e., the positive charge or hole) on the base [128]. This type of potential triple damage would also be created by a single electron and should be considered as a possible single-electron event leading to DSBs.

In conclusion, the hyperthermal reactions of Figure 3 can be triggered immediately following initial ionization by HER. Moreover, the DNA cluster lesions (D to F) that are potentially lethal to cells do not need to be created from multiple independent events [129]. Hence, in future modeling of the sequence of events triggered by HER that reduce cellular function, single collisions of secondary electrons causing cluster lesions to DNA should also be included.

## 5. Summary

We provide in Table 1 the first detailed and complete set of ACSs for 1–20 eV electron damage to DNA, including SSBs, BDs, CLs, BD, BD-CLs, DSBs, and NDCDs. Among them, SSBs and isolated BDs constitute the largest portion of the total damage. However, CLs, DSBs, and non-DSB cluster lesions produced in smaller numbers can be detrimental to cell functionality and survival. The electron energy dependence indicates that the maxima at 5 or 6 and 10 eV, appearing in all ACSs, arise from the decay of core-excited resonances into destructive channels. The rise at 2 eV is only observed for single lesions, including CLs, BDs, and SSBs. They are due to the decay of shape resonances into the DEA channel. The ACSs listed in Table 1 necessarily incorporate the quantum behavior of LEEs, which derives from the quasi-unidimensional band structure of DNA present at hyperthermal energies above the vacuum level. As seen from the DNA helix, the stacking of the bases at nearly equidistant relative positions is expected to produce a well-structured conduction band. These are reflected in the quantum behavior of 0–20 electrons and the ACSs.

The present results should stimulate the development of MC programs capable of incorporating ACSs, such as those provided in Table 1, and hence the inherent quantum mechanics of electron scattering within DNA. Such codes are expected to be particularly efficient for nanodosimetry, where the concept of local dose is more difficult to define. Further experimental efforts should therefore be made to investigate LEE scattering from and attachment to DNA surrounded by biomolecules found in the cell nucleus, with the perspective of generating damage ACSs from an environment emulating cellular DNA. The results could be combined with those of femtosecond-laser micro-irradiation of cells, which require such ACSs to determine the role of the induced low-energy photoelectron distribution in cell survival [130,131,132]. The combination of results of LEE thin-film and laser experiments thus shows considerable promise to probe the action of LEEs within living cells.

## Figures and Tables

**Table 1:** ACSs (×10^−15^ cm^2^) for crosslinks (CLs), DSBs, SSBs, loss of the supercoiled configuration (LS), BD-related crosslinks (BD-CLs), isolated BDs, non-DSB clustered damages (NDCDs), total BDs, and total DNA damages induced by 1–20 eV electrons in 3197 base-pair plasmid DNA. The last two lines are highlighted in color to indicate the ACSs for Arginine-DNA complexes induced by 5 and 10 eV electrons in the same plasmids under identical conditions. * Wang et al. [65].

Energy (eV)	CLs	DSBs	SSBs	LS	BD-CLs	Non-DSB Clustered Damages	Isolated	Total BDs	Total DNA
							BDs		Damages
1	1.7 ± 0.5	n.d.	31.6 ± 4.1	37.1 ± 6.1	1.5 ± 1.0	n.d.	37.1 ± 9.6	39.6 ± 12.4	76.7 ± 13.5
2	n.d.	n.d.	30.2 ± 3.1	34.7 ± 3.0	n.d.	n.d.	46.4 ± 9.2	45.5 ± 7.7	80.2 ± 9.4
3	1.0 ± 0.2	n.d.	23.9 ± 4.0	28.8 ± 3.9	0.5 ± 0.3	n.d.	18.7 ± 8.0	21.1 ± 6.9	49.9 ± 7.8
4	1.4 ± 0.3	1.2 ± 0.3	29.8 ± 3.1	36.2 ± 3.9	0.7 ± 0.6	0.7 ± 0.5	21.1 ± 5.7	25.5 ± 5.1	61.6 ± 6.8
5	2.1 ± 0.3	1.5 ± 0.3	37.5 ± 4.7	44.7 ± 5.8	1.1 ± 0.6	1.3 ± 0.8	22.9 ± 9.8	29.9 ± 13.3	74.6 ± 14.3
6	1.5 ± 0.2	2.3 ± 0.2	18.2 ± 2.2	23.8 ± 3.2	0.9 ± 0.5	1.6 ± 1.0	12.2 ± 3.9	12.7 ± 5.4	36.9 ± 5.5
7	1.5 ± 0.1	1.4 ± 0.5	29.5 ± 3.4	37.6 ± 4.7	0.6 ± 0.4	1.1 ± 0.7	23.4 ± 12.5	25.9 ± 12.5	65.5 ± 13.3
8	1.9 ± 0.3	1.6 ± 0.2	36.6 ± 4.2	42.8 ± 4.7	0.8 ± 0.5	1.2 ± 0.9	24.2 ± 4.2	27.9 ± 10.9	70.7 ± 12.1
9	2.0 ± 0.4	2.7 ± 0.6	39.3 ± 4.2	47.3 ± 6.1	1.5 ± 1.0	1.9 ± 1.2	28.2 ± 11.0	37.4 ± 15.3	84.7 ± 16.5
10	3.7 ± 0.8	3.5 ± 0.6	45.5 ± 4.1	52.0 ± 6.4	2.9 ± 1.1	5.1 ± 1.4	54.0 ± 16.4	51.5 ± 19.7	103.5 ± 21.0
11	2.3 ± 0.4	2.6 ± 0.5	35.1 ± 4.5	40.8 ± 5.1	1.1 ± 0.8	1.3 ± 0.8	25.6 ± 13.3	26.5 ± 11.1	67.3 ± 12.1
12	2.1 ± 0.4	2.1 ± 0.4	28.4 ± 2.6	35.3 ± 3.2	0.9 ± 0.4	1.2 ± 0.7	22.8 ± 8.9	23.9 ± 5.0	59.2 ± 6.6
13	1.7 ± 0.2	1.4 ± 0.5	27.7 ± 4.3	33.3 ± 4.7	1.2 ± 0.4	1.2 ± 0.7	19.1 ± 7.9	20.4 ± 6.5	54.6 ± 7.7
14	1.1 ± 0.2	1.3 ± 0.1	27.4 ± 6.9	29.9 ± 4.0	0.8 ± 0.1	0.8 ± 0.6	12.3 ±7.2	12.6 ± 2.9	42.5 ± 4.3
15	1.2 ± 0.2	1.4 ± 0.4	36.4 ± 3.2	41.2 ± 3.7	1.0 ± 0.2	0.7 ± 0.5	11.0 ± 5.4	12.5 ± 5.1	53.7 ± 6.6
16	1.6 ± 0.2	1.5 ± 0.3	38.2 ± 3.9	43.3 ± 7.8	1.1 ± 0.4	0.9 ± 0.6	9.9 ± 6.4	11.0 ± 3.3	54.3 ± 5.4
17	1.3 ± 0.2	1.4 ± 0.4	40.2 ± 3.8	43.3 ± 5.2	1.5 ± 0.3	1.2 ± 0.5	15.6 ± 5.8	18.3 ± 6.1	61.6 ± 7.7
18	1.7 ± 0.3	1.3 ± 0.3	40.6 ± 7.7	43.8 ± 5.1	1.4 ± 0.5	0.9 ± 0.7	19.0 ± 8.9	20.5 ± 8.1	55.3 ± 9.1
20	1.6 ± 0.3	1.6 ± 0.4	40.8 ± 3.8	44.7 ± 4.5	1.6 ± 0.6	1.1 ± 0.6	23.9 ± 5.3	24.7 ± 5.7	69.4 ± 7.7
5 * 10 *	3.9 ± 0.6	1.4 ± 0.2	8.4 ± 0.7	17.3 ± 0.3	0.8 ± 0.1	0.6 ± 0.3	23.0 ± 0.3	26.5 ± 1.1	45.8 ± 0.7
	5.7 ± 0.6	2.7 ± 0.8	12.3 ± 1.2	20.0 ± 1.0	4.3 ± 1.9	3.4 ± 2.0	28.2 ± 2.9	35.1 ± 2.3	57.5 ± 2.8

## Data Availability

The original contributions presented in this study are included in the article/supplementary material. Further inquiries can be directed to the corresponding author.

## Supplementary Materials

The following supporting information can be downloaded at: https://www.mdpi.com/article/10.3390/ijms26094057/s1.

## Abbreviations

ACS	Absolute cross section
Arg-DNA	Arginine-DNA
BD	Base damage
BD-CLs	Base damage related CLs
BER	Base excision repair
CL	Crosslink
CS	Cross section
DEA	Dissociative electron attachment
DSB	Double strand break
HER	High energy radiation
LEE	Low-energy electron
LS	Loss of supercoiled
MC	Monte Carlo
NDCD	Non-DSB cluster damages
PF	Protection factor
SE	Secondary electron
SSB	Single strand break
TA	Transient anion
OND	Other non-detected
