# Supplementary material for: Hyperthermal Reactions in DNA Triggered by 1–20 eV Electrons: Absolute Cross Sections for Crosslinks, Strand Breaks, Clustered Damages and Base Modifications
Source: Int J Mol Sci. 2025 Apr 25;26(9):4057. doi: 10.3390/ijms26094057 (PMC12072190; doi:10.3390/ijms26094057)
Supplement: Supplementary file 1 [file ijms-26-04057-s001.zip › ijms-3564632-supplementary.pdf]

Supplementary Materials

Hyperthermal reactions in DNA triggered by 1-20 eV electrons: Absolute cross sections for crosslinks, strand breaks, clustered damages and base modifications

Yanfang Dong,[a] Xin Huang,[b] Wenlu Zhang,[a] Yu Shao,[b] Pierre Cloutier,[c] Yi Zheng[b,c] and Léon Sanche[c]*

[a]College of Basic Medicine and Forensic Medicine, Henan University of Science and Technology, Luoyang, 471000, P.R.China; [b]State Key Laboratory of Photocatalysis on Energy and Environment, Faculty of Chemistry, Fuzhou University, Fuzhou 350116, P.R. China; [c]Department of Medical Imaging and Radiation Sciences, Faculty of Medicine and Health Sciences, Université de Sherbrooke, Sherbrooke, QC Canada J1H 5N4

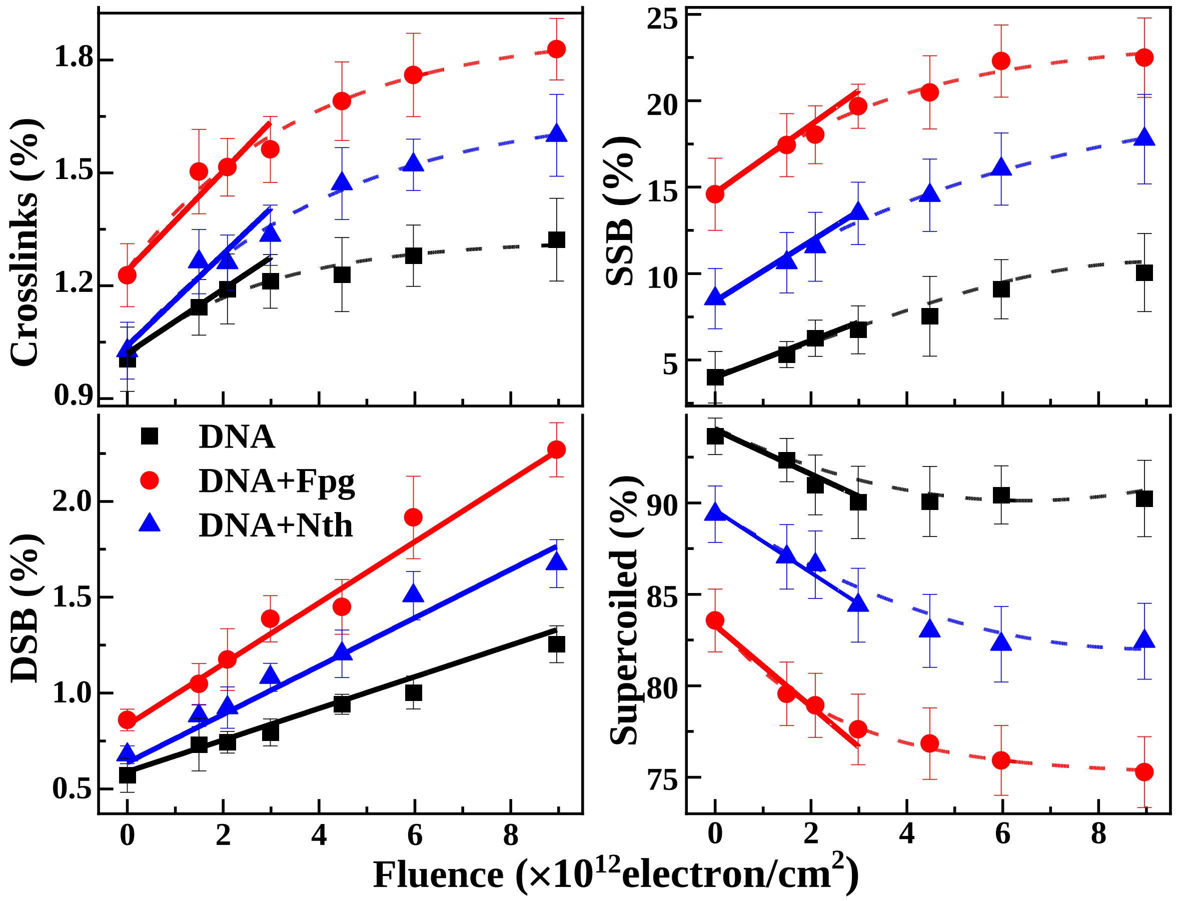

Figure S1 Fluence–response curves for the percentage yields of CLs, SSBs, and DSBs and loss of the supercoiled configuration in DNA films (■) deposited by lyophilization. The damage was induced by 10 eV electrons. Percentage yields after treatment with Fpg (red ●) and Nth (blue ▲) enzymes are represented by the red and blue curves, respectively. The dashed lines are exponential fits and the solid lines the initial slopes. The exponential behavior is due to the depletion of the initial number of supercoiled DNA with fluence. Both supercoiled DNA, and the linear form created by a SSB, can serve as an initial source for DSBs. This source remains essentially constant with fluence and hence the DSB response curve can be fitted to a linear function. Each data point is the result of eight identical bombardment procedures, and the error bars are the standard deviations.

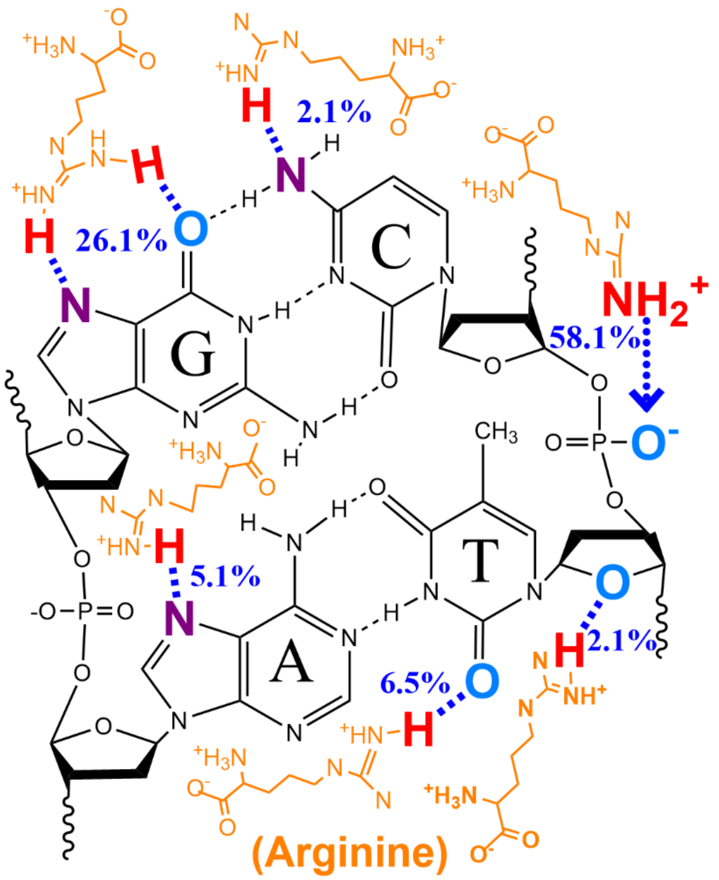

Figure S2 Binding sites of Arginine to DNA. The numbers in blue indicate the binding percentage taken from references [1,2]. Hydrogen bonds are represented by blue dotted lines, while the blue arrow refers to the electrostatic force between O^- and the amino group. Arginine molecules are shown in orange. Copyright 2023 American Chemical Society.

Table S1 Comparison of absolute cross sections (ACSs) for 10 eV electrons to induce conformational damage (i.e., loss of the initial supercoiled configuration) in 3,197 base-pair plasmid DNA. *f* is the penetration factor applied to the measured effective cross sections (CSs) to generate the ACSs. σ_n corresponds the cross section (CS) per nucleotide.

Reference	Film Preparation Method	Electron Energy (eV)	Film Thickness (nm)	*f*	σ (10^{-14}cm^2)	σ_n (10^{-18}cm^2)
Chen et al. [3]	Lyophilization	9.6	10	0.72 ± 0.15	5.1 ± 1.3	8.0 ± 2.1
			15	0.62 ± 0.18	5.1 ± 1.8	8.0 ± 2.8
			20	0.53 ± 0.19	5.0 ± 2.6	7.8 ± 4.0
Rezaee et al. [4]	Lyophilization	10	10	0.6 ± 0.18	3.7 ± 1.4	5.8 ± 2.2
			15	0.5 ± 0.28	3.9 ± 1.5	6.1 ± 2.3
			20	0.4 ± 0.23	3.6 ± 1.4	5.6 ± 2.2
Boulanouar et al. [5]	Self-assembly	10	8.8, 15.3, 19.8	0.73 ± 0.17	3.0 ± 0.3	4.8 ± 0.5
Brodeur et al. [6]	Self-assembly	10	10.5 ± 3.3	0.61 ± 0.19	4.7 ± 1.5	7.4 ± 2.3

Table S2 ACSs (×10^{-15} cm^2) of DNA damages for Arg-DNA induced by 5 and 10 eV electron and protection factors (PFs) taken as the ratio of the ACS of pure DNA to that of Arg−DNA.

$PF=\sigma_{DNA}/\sigma_{mol\text{-}DNA}$	E(eV)	CLs	DSBs	SSBs	LS	BD-CLs	Non-DSB clustered damages	Isolated BDs	Total BDs	Total DNA damage
$CS_{(Arg)}$ [7]	5	3.9 ± 0.6	1.4 ± 0.2	8.4 ± 0.7	17.3 ± 0.3	0.8 ± 0.1	0.6 ± 0.3	23.0 ± 0.3	26.5 ± 1.1	45.8 ± 0.7
	10	5.7 ± 0.6	2.7 ± 0.8	12.3 ± 1.2	20.0 ± 1.0	4.3 ± 1.9	3.4 ± 2.0	28.2 ± 2.9	35.1 ± 2.3	57.5 ± 2.8
$PF_{(Arg)}$	5	0.5 ± 0.1	1.1 ± 0.3	4.5 ± 0.7	2.6 ± 0.3	1.4 ± 0.8	2.1 ± 1.7	1.0 ± 0.4	1.1 ± 0.5	1.6 ± 0.3
	10	0.6 ± 0.2	1.3 ± 0.4	3.7 ± 0.5	2.6 ± 0.3	0.7 ± 0.4	1.5 ± 1.0	1.9 ± 0.6	1.5 ± 0.6	1.8 ± 0.4
$PF_{(Arg)}$ [7]	5	1.2 ± 0.2	1.1 ± 0.2	4.4 ± 0.1	2.5 ± 0.1	1.4 ± 0.6	2.0 ± 0.8	1.0 ± 0.4	1.1 ± 0.5	1.6 ± 0.2
	10	1.3 ± 0.1	1.6 ± 0.3	3.7 ± 0.4	2.6 ± 0.3	1.5 ± 1.1	1.9 ± 1.2	1.8 ± 0.5	1.6 ± 0.3	1.8 ± 0.2

References

1. Luscombe, N.M.; Laskowski, R.A.; Thornton, J.M. Amino acid-base interactions: A three-dimensional analysis of protein-DNA interactions at an atomic level. *Nucleic. Acids. Res.* **2001**, *29,* 2860-2874.
2. Cheng, A.C.; Frankel, A.D. Ab initio interaction energies of hydrogen-bonded amino acid side chain-nucleic acid base interactions. *J. Am. Chem. Soc.* **2004**, *126,* 434-435.
3. Chen, W.; Chen, S.; Dong, Y.; Cloutier, P.; Zheng, Y.; Sanche, L. Absolute cross-sections for DNA strand breaks and crosslinks induced by low energy electrons. *Phys. Chem. Chem. Phys.* **2016**, *18,* 32762-32771.
4. Rezaee, M.; Cloutier, P.; Bass, A. D.; Michaud, M.; Hunting, D. J.; Sanche, L. Absolute cross section for low-energy-electron damage to condensed macromolecules: A case study of DNA. *Phys. Rev. E.* **2012**, *86*, 031913.
5. Boulanouar, O.; Fromm, M.; Bass, A. D.; Cloutier, P.; Sanche, L. Absolute cross section for loss of supercoiled topology induced by 10 eV electrons in highly uniform /DNA/1,3-diaminopropane films deposited on highly ordered pyrolitic graphite. *J. Chem. Phys.* **2013**, *139,* 055104.
6. Brodeur, N.; Cloutier, P.; Bass, A. D.; Bertrand, G.; Hunting, D. J.; Grandbois, M.; Sanche, L. Absolute cross section for DNA damage induced by low-energy (10 eV) electrons: Experimental refinements and sample characterization by AFM. *J. Chem. Phys.* **2018**, *149*, 164904.
7. Wang, X.; Liao, H.; Liu, W.; Shao, Y.; Zheng, Y.; Sanche, L. DNA protection against damages induced by low-energy electrons: absolute cross sections for arginine-DNA complexes. *J. Phys. Chem. Lett.* **2023**, *14*, 5674-5680.
